# Sexual Health and the Internet: Cross-Sectional Study of Online Preferences Among Adolescents

**DOI:** 10.2196/jmir.7068

**Published:** 2017-11-08

**Authors:** Antonella Juline von Rosen, Frederik Tilmann von Rosen, Peter Tinnemann, Falk Müller-Riemenschneider

**Affiliations:** ^1^ Institute for Social Medicine, Epidemiology and Health Economics Charité – Universitätsmedizin Berlin Berlin Germany; ^2^ Institute for Public Health and Nursing Research University of Bremen Bremen Germany; ^3^ Saw Swee Hock School of Public Health National University of Singapore Singapore Singapore

**Keywords:** adolescent, adolescent behavior, Internet, reproductive health, health literacy, sex education, cross-sectional studies, online preferences, eHealth literacy

## Abstract

**Background:**

The Internet is widely used by adolescents for sexual health information and bears the potential to increase knowledge and positively affect behavior.

**Objective:**

The objective of this study is to assess students’ preferences when looking for sexual health information online.

**Methods:**

We conducted a cross-sectional survey among ninth grade students in a convenience sample of 13 secondary schools in Berlin, Germany. During a regular school period, participants were requested to rate the importance they attribute to nine aspects of sexual health websites in a paper-based questionnaire. Bivariate and multivariable analyses were used to assess awareness and preferences by gender, age, migrant background, and school type.

**Results:**

Of 1190 eligible students, 1177 (98.91%) students with a mean age of 14.6 (SD 0.7) years participated, 52.52% (605/1152) were male, and 52.94% (612/1156) had at least one parent born abroad. Participant numbers were spread equally across three types of secondary schools in Berlin. Website aspects most frequently cited as important were easily comprehensible wording (88.33%, 961/1088), clear information layout (80.57%, 871/1081), and reliability of the website’s publisher (79.28%, 857/1081), whereas the visual style of a website was deemed important by the lowest number of students (35.13%, 378/1076). There was a marked gender difference in the importance students attached to website publisher reliability. Although 437/515 (84.9%) of female participants regarded this as important, only 420/566 (74.2%) of male participants did likewise (*P*<.001). In multivariable analyses, demographic differences were also particularly visible in the importance of publisher reliability: male participants were significantly less likely to find this aspect important (OR 0.50, 95% CI 0.37-0.69). The odds ratio for students with migrant background was 0.64 (95% CI 0.50-0.81, reference=no migrant background) and OR 2.04 (95% CI 1.03-4.03) for students in the most academic school type (reference=least academic).

**Conclusions:**

Students prefer easily understandable online resources. Setting up sexual health websites according to the explicit preferences of the target audience might encourage usage, especially by those subpopulations less likely to critically assess information validity: male adolescents, children of immigrants, and the academically disadvantaged.

## Introduction

### The Internet as a Source of Sexual Health Information

The rapid expansion of the Internet [1,2] has led to an information revolution that affects nearly all aspects of our lives. It completely changed the way we access, analyze, and use information. This holds true even for areas as delicate and private as health. In the United States, searching for health information became the third most popular online activity for all Internet users in 2010 [3]. Several studies show that the vast majority of adolescents look for health information online [4-6]. Of particular interest for teenagers is sexual health, an area raising a multitude of questions perceived as embarrassing, controversial, or sensitive [5-9]. The threshold for adolescents to search for answers to these questions is lowered by the anonymous nature of the Internet, its easy and quick availability, and its low cost [10,11]. A digital divide has been widely described, with socioeconomically disadvantaged subpopulations less likely to have access to the Internet and to use it regularly [12]. However, recent evidence shows that the divide regarding Internet access is closing in industrialized countries, especially among adolescents and young adults. A representative survey in Germany in 2016 showed that across socioeconomic and ethnic divides, 100% of respondents aged 16 to 24 years had Internet access [13], with 97% stating daily usage [14].

The Internet hence bears the potential to increase sexual health knowledge and promote healthy behavior, especially in socioeconomic and ethnic groups with lower levels of access to traditional sources of sexual health information [5,15]. However, reputable sexual health websites with sound content coexist with a vast array of websites presenting incorrect or incomplete information that can misinform and might ultimately lead to unhealthy behavior [16,17]. In the competitive market for online patronage, user centricity has been suggested as an important advantage [18]. Providers of sexual health information, such as public health agencies, should strive to offer online resources that closely match the requirements and preferences of their target population.

To date, evidence on adolescents’ health website preferences is scarce and exclusively qualitative. The sole study focusing specifically on adolescents’ evaluation of a sexual health website found that interviewees frequently disliked text-heavy style and appreciated interactive features and “real-life stories” from other adolescents [19]. A study on adolescents’ evaluation of a general health website yielded similar results [20]. A study on adolescents’ website preferences, albeit with a mental health focus, concluded that interviewees were highly critical of website publisher credibility, and that academically disadvantaged participants in particular preferred low text density [21].

To our knowledge, no quantitative study design has to date been employed to assess adolescents’ sexual health website preferences. Although qualitative research is clearly useful in gaining an in-depth understanding of preferences, a quantitative study can offer insight into preferences of a wider sample and highlight and quantify differences between different subpopulations.

### Study Objective

The objective of this study is to assess which aspects of a website adolescents deem important when they look for sexual health information online, and to assess differences between demographic subpopulations.

## Methods

### Study Design

Data were collected as part of a larger cross-sectional study on sexual health knowledge that was conducted throughout the year 2012 in the ninth grades of secondary schools in Berlin, Germany. The study, its methodology, and participant demography have been partially described elsewhere [22].

The study design was approved by the Ethics Committee of the Charité – Universitätsmedizin Berlin, as well as the Berlin Senate’s Department for Education, Youth and Science. In accordance with legal requirements, the study was discussed and approved by parent-teacher conferences in all schools prior to study onset. As demanded by state law, students aged 13 years and younger were required to provide written parental consent.

Reporting on this study was based on the Strengthening the Reporting of Observational Studies in Epidemiology (STROBE) statement guidelines [23].

### Sampling and Data Collection

The project study group contacted the heads of the biology or natural science departments of all public secondary schools by phone in late 2011 and supplied schools with further information on the study via email. Of a total of 287 eligible schools, the heads of department were successfully contacted in 142 cases, with 13 schools agreeing to participate. In all 13 schools, the parent-teacher conferences voted in favor of participation. Of the schools choosing not to partake, all quoted time constraints of teaching staff as the reason.

Since a major education reform implemented in 2010, state secondary education in Berlin formally consists of two school forms: university-preparatory schools [Gymnasium] and Integrated Secondary Schools [Integrierte Gesamtschule]. An important subdivision can be made in the latter category between schools allowing for their highest-achieving students to continue up to year 12 to qualify for university access, and schools where no such option exists and most students are likely not to continue their formal education past year 10. For brevity, these three school types were reported in results as “highest (academic) tier” (gymnasium), “intermediate (academic) tier” (integrated secondary school with option to qualify for university), and “lowest (academic) tier” (integrated secondary school up to year 10 only). All three school types were represented in the sample, as were seven of the 12 Berlin city districts (Mitte, Pankow, Charlottenburg-Wilmersdorf, Spandau, Steglitz-Zehlendorf, Treptow-Köpenick, and Marzahn-Hellersdorf). Districts from both the former Eastern and Western parts of Berlin were included, and districts with participating schools ranged from inner city to suburban and from the most affluent to relatively deprived [24].

### Study Setting

The survey was conducted by a member of the project team during a school period in the regular classroom setting. A short presentation informed students on the aim of the study and of the voluntary and anonymous nature of participation. It was emphasized that results would not be disclosed to teaching staff, parents, or fellow students. To safeguard anonymity, it was stressed that it was strictly prohibited to attempt to read other students’ responses, and an exam-like, nonclustered seating layout in the classroom was adopted. Subsequently, a paper-based questionnaire was administered to all participating students. A member of the research team supervised the class until all questionnaires had been collected.

### Questionnaire

Due to the lack of existing tools for the research question, the study questionnaire was devised by the authors to assess students’ preferences and requirements when looking for sexual health information online. To ascertain the adequacy of the questionnaire for the adolescent sample population, a pretest was performed in one school class, with comments from students leading to minor modifications. Following discussion with students from the pretest sample, wording was phrased indirectly (“How important would you find the following aspects when looking for sexual health information on the Internet?”). Importance was rated on a five-point Likert scale, on which students were to choose between “important,” “somewhat important,” “neither important nor unimportant,” “rather unimportant,” and “unimportant” (authors’ translation from German).

Demographic variables on gender, age, and migrant background (parental place of birth) were included in the questionnaire. For the latter, response options were restricted to “Germany” or “other country” in accordance with the principle of parental informational self-determination.

Aspects were chosen by the authors following an in-depth analysis of the different characteristics and features of 10 prominent German-language websites providing sexual health information: Loveline [25], Mach’s mit [26], profamilia [27], Sexundso [28], sextra [29], BRAVO [30], gutefrage [31], mädchen.de [32], gofeminin [33], and Lovetalk [34]. Websites were chosen based on a search on Google for the following keywords and combinations thereof: adolescents, students, love, sex, first time, contraception, condom, pill, pregnancy, STIs, and HIV (authors’ translation of the German keywords). Google was used as the most frequently cited starting point for sexual health information online in previous research [35]. A total of nine aspects belonging to three principal groups were identified: “who and for whom” (publisher and explicit target audience of a website), “information presentation” (visual style, clear layout of information, easily understandable language, and text shortness), and “website features” (facilities to ask individual questions, a section where people can report their own personal experiences, and advice provided by people of similar age). The websites as well as the nine website aspects were discussed with and deemed appropriate by our pretest sample.

### Statistics and Data Analysis

For the analysis of the survey data, IBM SPSS version 23 was used. Descriptives were computed. For the statistical analyses, outcomes were dichotomized for the sake of readability: the variables “important” and “rather important” were clustered to “(rather) important,” as were the categories “of medium importance,” “rather unimportant,” and “unimportant” to “other response.” Descriptives and chi-square tests for the original five-point outcome scale are included in Multimedia Appendix 1.

### Descriptives and Bivariate Analysis

Aspects were ranked by the aggregate percentage of students selecting “(rather) important.” Using two-sided chi-square tests, bivariate relationships were calculated between the demographic variables age, gender, migratory background, and school type and the outcome variables measuring the importance participants attribute to the different aspects of sexual health information websites. For statistical analysis, migrant background was defined as having at least one parent who was born abroad.

### Multivariable Analysis

Regression models were used to quantify the effect of age, gender, migratory background, and school type on outcome variables. Due to the clustered nature of observation in class and school groups, a mixed multilevel regression model (SPSS GENLINMIXED) containing school and class as random effects was used. Odds ratios and 95% confidence intervals for all outcome variables were computed from mixed multilevel regression. To account for possible violations of model assumptions, we used robust estimation. Missing cases were excluded from bivariate and regression analysis.

## Results

### Population

The study was conducted in 61 school classes. A total of 1190 students were present in class on the day of the study and thus eligible for participation. Ten students aged 13 years could not participate due to missing parental consent and a further two students chose not to participate. One student could not participate due to lacking the required basic language proficiency. Therefore, a total of 1177 participants were included in the survey, equating to a response rate of 98.91%. The overall mean age of participants was 14.6 (SD 0.8) years, the mean for boys being 14.6 (SD 0.7) years and 14.5 (SD 0.7) years for girls. Table 1 shows the demography of study participants, as reported previously [22].

### Descriptives and Bivariate Analysis

Table 2 shows the distribution of responses for the items in the three groups of website aspects, the relative rank of each aspect by cumulative percentage selecting “(rather) important,” and stratification by gender.

**Table 1:** Demography of participants (N=1177).

Population characteristic		n (%)
**Age (years)**		**n=1162^a^**
	13	23 (1.98)
	14	565 (48.62)
	15	480 (41.31)
	16	94 (8.09)
**Gender**		**n=1152^a^**
	Female	547 (47.48)
	Male	605 (52.52)
**Migrant background**		**n=1156^a^**
	Both parents born in Germany	544 (47.06)
	Both parents born abroad	352 (30.45)
	Only mother born abroad	127 (10.99)
	Only father born abroad	133 (11.51)
**School type**		**n=1177^b^**
	Lowest tier	390 (33.14)
	Intermediate tier	395 (33.56)
	Highest tier	392 (33.31)

^a^Total number of responses for each variable.

^b^Entered for all participants at school level.

**Table 2:** Website preferences by gender (bivariate analysis).

Website aspect group and website aspect		Aspect rank^a^	Population in (rather) important, n (%)			*P*^b^
			Female	Male	Total	
**Who and for whom**						
	Reputable publisher (n=1081^c^)	3	437 (84.85)	420 (74.20)	857 (79.28)	<.001
	Explicitly addressed at adolescents (n=1088^c^)	7	366 (70.66)	311 (54.56)	677 (62.22)	<.001
**Information presentation**						
	Language easily understandable (n=1088^c^)	1	463 (89. 38)	498 (87.37)	961 (88.33)	.35
	Information clearly laid out (n=1081^c^)	2	425 (83.17)	446 (78.25)	871 (80.57)	.045
	Texts short and concise (n=1084^c^)	8	260 (50.58)	304 (53.33)	564 (52.03)	.39
	Visual style/design attractive (n=1076^c^)	9	149 (29.22)	229 (40.46)	378 (35.13)	<.001
**Website features**						
	Possibility to ask questions (n=1079^c^)	4	378 (74.14)	406 (71. 35)	784 (72.66)	.34
	Section with personal experiences (n=1080^c^)	5	361 (70.23)	381 (67.31)	742 (68.70)	.32
	Advice by other adolescents (n=1072^c^)	6	336 (66.14)	373 (66.13)	709 (66.14)	>.99

^a^By percentage (rather) important.

^b^Calculated from chi-square tests.

^c^Number of participants included in the analysis.

### Student Preferences: Who and for Whom

Publisher reliability was described as (rather) important by 857 of 1081 students (79.28%), the percentage for girls being higher than for boys. Students of migrant background were significantly less likely to attach importance to this aspect (*P*<.001), as were students in the lowest academic tier of schools (*P*=.01). Whether a website is explicitly addressed at adolescents was an aspect to which students attributed relatively little importance. Again, the aspect was considerably more important to girls, although there was no association with the other demographic variables.

### Student Preferences: Information Presentation

Understandable language was the most important aspect overall. Importance was higher in the highest academic school tier (*P*=.001) and lower for children of immigrants (*P*<.001). Across the sample, 871 of 1081 respondents (80.57%) described it as (rather) important that information should be clearly laid out. Girls were significantly more likely than boys to regard this aspect as (rather) important. Again, school type and migratory background were significantly associated with outcomes: the higher the school tier, the more important the issue was considered (*P*=.03), and children of immigrants were less likely to regard the aspect as (rather) important (*P*<.001).

Whether information text on sexual health websites was short and concise was an issue of relatively low importance in our survey, with 564 of 1084 students (52.03%) attaching importance. Students from the lowest tier of schools and children of immigrants were significantly more likely to regard this aspect as (rather) important (*P*=.01 for both).

Visual style was the least important issue overall. Although no significant difference by migratory background was observed, school type was significantly associated with the importance attached to a website’s visual style: students from the lowest tier of schools were significantly more likely to regard this aspect as (rather) important (*P*=.01).

### Student Preferences: Website Features

Among participating students, 784 of 1079 (72.66%) found it (rather) important that sexual health websites offer the possibility to ask individual questions, thus putting it in fourth place overall. It was followed in fifth place by the aspect that websites should include a section where people can report their own personal experiences and in sixth place by whether websites included advice written by people of similar age as the user. Students with migrant background were less likely to deem personal experiences and writing by age peers (rather) important (*P*=.04 and *P*=.03, respectively). Otherwise, for website feature variables, no significant difference by gender, age, migratory background, or school type was observed.

### Multivariable Analysis

Results from the regression model are presented in Table 3. Differences in odds were especially marked on the issue of the reputability of a website’s publisher: female students were nearly twice as likely to attach importance to this aspect, with not having a migrant background and attending a school in the highest academic tier also greatly increasing the odds to select this option. Being of female gender also increased the odds of attributing importance to whether a website was explicitly addressed at adolescents by more than twofold.

**Table 3:** The effect of the demographic variables on outcomes (multivariable analysis).

Aspect group and website aspect		Variable, OR (95% CI)^a^				
		Age (per year increase)	Gender (male)	Migration (migrant background)	School type (intermediate tier)	School type (highest tier)
**Who and for whom**						
	Reputable publisher (n=1074^b^)	1.23 (1.03-1.47)^c^	0.50 (0.37-0.69)^c^	0.64 (0.50-0.81)^c^	1.39 (0.70-2.73)	2.04 (1.03-4.03)^c^
	Explicitly addressed at adolescents (n=1081^b^)	1.16 (0.96-1.40)	0.48 (0.35-0.67)^c^	0.76 (0.66-0.88)^c^	1.08 (0.74-1.57)	1.02 (0.83-1.26)
**Information presentation**						
	Language easily understandable (n=1081^b^)	1.02 (0.91-1.13)	0.85 (0.51-1.39)	0.47 (0.29-0.75)^c^	1.33 (0.70-2.53)	2.62 (1.36-5.06)^c^
	Information clearly laid out (n=1074^b^)	1.13 (1.01-1.27)^c^	0.71 (0.48-1.06)	0.71 (0.48-1.04)	1.10 (0.42-2.86)	1.75 (0.85-3.59)
	Texts short and concise (n=1077^b^)	1.32 (1.10-1.58)^c^	1.02 (0.84-1.25)	1.46 (1.10-1.94)^c^	0.71 (0.50-1.03)	0.81 (0.46-1.44)
	Visual style/design attractive (n=1069^b^)	1.15 (1.00-1.32)^c^	1.57 (1.08-2.29)^c^	1.02 (0.78-1.32)	0.68 (0.52-0.90)^c^	1.07 (0.70-1.52)
**Website features**						
	Possibility to ask questions (n=1072^b^)	1.00 (0.82-1.24)	0.87 (0.71-1.07)	0.89 (0.64-1.24)	1.06 (0.71-1.58)	1.14 (0.80-1.62)
	Section with personal experiences (n=1073^b^)	1.17 (1.02-1.33)^c^	0.84 (0.63-1.14)	0.88 (0.69-1.11)	0.87 (0.48-1.60)	1.17 (0.68-2.01)
	Advice from other adolescents (n=1065^b^)	1.08 (0.06-8.62)	0.99 (0.76-1.23)	0.78 (0.59-1.04)	0.91 (0.61-1.37)	1.27 (0.92-1.75)

^a^Reference category for gender was female, migration was none, and for both school types was lowest tier.

^b^Number of participants included in the analysis.

^c^*P*<.05.

## Discussion

We conducted a study among ninth graders in Berlin secondary schools to evaluate adolescents’ preferences when looking for sexual health information online. Despite the voluntary nature of participation, only two of 1179 students chose not to participate. The overwhelming rate of participation might be an expression of a strong interest and curiosity invoked by the topic of sexual health and Internet research among adolescents. To our knowledge, no quantitative study with a similar focus on adolescent preferences in online resources has been conducted to date.

Easily comprehensible wording was most frequently selected as (rather) important. This was followed by clear information layout. In third place came the credibility of a website’s publisher, which was described as (rather) important by about 80% of participants. We found that male gender, migratory background, and attending a school of the lowest academic tier were significantly associated with lower importance placed on publisher reliability in both bivariate and multivariable analyses. The possibility to ask questions, whether websites contained a section with personal experiences, and whether websites included advice from other adolescents were of intermediate relative importance to students in our sample. Interestingly, results for website feature aspects were relatively uniform across demographic divides. Both the visual style and the youth specificity of websites were relatively unimportant to study participants. Male respondents were more likely to regard this aspect as (rather) important.

The Internet has enabled direct access to a vast array of health information, which was previously only available through intermediates such as health care professionals. The term “disintermediation” has been coined to describe this development [36]. It promises greater access to health information, especially in sensitive areas such as sexual health [9]. However, it also bears the risk that incorrect information is accessed and perceived as accurate [17,37]. This is emphasized by the visible minority of students in our sample neglecting publisher credibility of sexual heath websites. From a public health perspective, a dual strategy should be employed to facilitate the access of adolescents to reliable sexual health information online. Firstly, health care professionals and educators can provide a certain degree of apomediation. Secondly, access of reliable and suitable sexual health information resources should be encouraged by adapting websites to explicitly cater to the target audience’s preferences, and by educating adolescents to critically access the validity of sexual health information online.

Apomediation describes the process in which individuals or institutions provide guidance in the digital sphere, for example by recommending particular websites [36]. One study has suggested that health care professionals can play a pivotal role in providing a list of suitable and reliable health websites as a first port of call [38]. The reach of such an intervention among adolescents might be even higher in Germany than in other contexts given that statutory health insurance covers and promotes a one-time routine checkup visit to a physician for all youths aged 12 to 14 years [39]. Thus, physicians can extend their reach to healthy teenagers, and access is not impeded by financial considerations for adolescents or parents. A similar role with even greater reach could be played by schools. Peer apomediation, such as other adolescents recommending sexual health resources through social media, also bears the potential to spread knowledge regarding sexual health resources online. Further studies are needed to evaluate the reach and effectiveness of professional and peer apomediation.

Although recommendations of trustworthy websites by health care professionals, teachers, and possibly peers could play a role in providing adolescents with websites to start their search for sexual health information, it has to be taken into account that many users access health information including sexual health information starting with search engine queries rather than through apomediation [35,40,41]. User centricity is a key factor in preventing consumers from quickly “bouncing” back to search results [18]. Especially in the field of health where incorrect or misleading information can undermine healthy behavior, it is imperative that websites by reputable providers of sexual health information are set up according to the preferences and requirements of users. Our study can contribute to an understanding of adolescent preferences in sexual health websites. The most frequently cited aspect in our study, easily comprehensible language, should be actively pursued in the development of all sexual health resources. Beyond matching their preferences, it is axiomatic that information that users can linguistically comprehend is more likely to improve knowledge and potentially encourage healthy behavior. However, studies show that many health websites fall short in this regard and employ a language prohibitively sophisticated for many users, especially the young and/or educationally disadvantaged [42,43]. Our results likely reflect similar experiences of participants’ with overly complicated sexual health information online. Sexual health websites should hence employ accessible language as a dually user-centric measure: to make a website both more used by and more useful to adolescents.

One way to further user centricity is to invite target group participation at the different steps of development [19]. Because our study shows significant divergence between preferences even within a narrow age bracket, it is critical that participation is invited from adolescents across genders and ethnic and educational backgrounds. It can be hypothesized that reputable sexual health information providers, such as public health authorities, government bodies, or sexual health associations, could use this as a competitive advantage: their bona fide standing is likely to make it easier to co-opt youths to participate in website development, such as through schools.

In some cases, differences between demographic groups might warrant the setting up of separate resources. For example, our study shows pronounced differences in preferences between male and female adolescents. One solution would be to develop sexual health websites aimed at one gender only, or to set up male and female subsites to be selected by users within one sexual health website. Indeed, a previous study has shown that a majority of adolescents would prefer gender-stratified sexual health websites [20].

However, there are many cases in which it will not be possible to develop separate resources for subpopulations with divergent preferences. One innovative way to make online health resources palatable to a diverse target audience is to enable users to interactively modify different parameters of a website [44]. For example, users could select between a text-heavy and a more visual presentation of information or between elaborate and short articles. This enables users to adapt the website not only to general personal preferences, but also according to the specific search context and purpose. This innovative and user-centric concept has been implemented and positively evaluated for a general health information database [44].

Lastly, it is important that adolescents are educated and encouraged to access, comprehend, and critically evaluate online health information and sexual health information. The term “eHealth literacy” has been coined for this set of skills [45-47]. An integral part of eHealth literacy is the ability to appraise the validity of online information [48]. In our study, approximately 80% of students described the reliability of a website’s publisher as (rather) important. This is consistent with previous studies that found the majority of users are critical when looking for health information online, whereas a visible minority tend to neglect the issue of credibility of health websites [49-51]. We found that male gender, migratory background, and attending a school of the lowest academic tier were associated with lower importance placed on publisher reliability. With strong evidence that these groups are also more likely to have poor sexual health knowledge and to engage in risk-taking behavior both in Germany [15] and in other settings [52,53], it is crucial to educate students to critically evaluate the reliability of information resources, especially in these vulnerable subpopulations. Particularly in countries with mandatory school education, such as Germany, schools could be instrumental in providing health and sexual health online literacy education to virtually all adolescents. For this, the significant institutional inertia of school policy has to be overcome and joint initiatives at the different levels of school policy are called for [54].

A key strength of this study is the large study population including students from all types of public schools in Berlin. Furthermore, a voluntary participation rate of 98.9% of students present in class makes it unlikely that participation/ nonparticipation introduced a significant bias.

Unlike the student level, in which the rate of participation was very high, it has to be acknowledged that less than 5% of Berlin secondary schools participated. Although a majority of Berlin’s city districts and very diverse academic, socioeconomic, and geographic settings were represented in the sample, the generalizability of results might be limited by a systematic difference between participating and nonparticipating schools. Furthermore, it should be considered that findings of adolescents in Berlin might not be generalizable to other geographic settings.

A further limitation of this study is the fact that students were asked to evaluate the importance of different website aspects when looking for sexual health information online, without reference to specific websites during the survey. The abstract thinking this requires might have been challenging for at least some of the students in the sample. Future studies could include a more concrete evaluation of website aspects. For example, qualitative methods such as focus group interviews could be employed to have students evaluate different sexual health websites or to gain a more in-depth understanding of by what means and how accurately students evaluate publisher reliability. Furthermore, website analytics could be employed to quantify how successfully different sexual health website formats are in attracting and retaining visitors.

Websites providing sexual health information for adolescents bear the potential to improve sexual health knowledge and promote healthy behavior. To be effective, websites should be set up according to the preferences and requirements of the target population not merely regarding content, but also regarding the way information is presented. Divergent preferences might warrant the establishment of websites specifically geared at specific subpopulations, such as male adolescents or adolescents with a migrant background. Furthermore, parents, schools, and public health authorities should strive to improve online literacy among adolescents to make sure they have the facilities to critically evaluate the reliability of information online—in all aspects of information access, but especially in the critical field of sexual health.

## Appendix

Multimedia Appendix 1 Unclustered Likert-Scale outcomes of website preferences by gender.
