# Supplementary material for: Sexual Health and the Internet: Cross-Sectional Study of Online Preferences Among Adolescents
Source: J Med Internet Res. 2017 Nov 8;19(11):e379. doi: 10.2196/jmir.7068 (PMC5700403; doi:10.2196/jmir.7068)
Supplement: Multimedia Appendix 1 [file jmir_v19i11e379_app1.pdf]

Multimedia Appendix 1. Unclustered Likert-Scale outcomes of website preferences by gender.

Aspect Group	Website Aspect	Population	Response important n(%)	rather imp. n(%)	neither/nor n(%)	rather unimp. n(%)	unimportant n(%)	P [b]
**Who and for whom**	Reputable publisher (n=1081[a])	Female	300 (58.25)	137 (26.60)	48 (9.32)	10 (1.94)	20 (3.88)	<.001
		Male	284 (50.18)	136 (24.03)	85 (15.02)	32 (5.65)	29 (5.12)	
		Total	584 (54.02)	273 (25.25)	133 (12.30)	42 (3.89)	49 (4.53)	
	Explicitly addressed at adolescents (n=1088[a])	Female	202 (39.00)	164 (31.66)	102 (19.69)	24 (4.63)	26 (5.02)	<.001
		Male	197 (34.56)	114 (20.00)	146 (25.61)	68 (11.93)	45 (7.89)	
		Total	399 (36.67)	278 (25.55)	248 (22.79)	92 (8.46)	71 (6.53)	
**Information Presentation**	Language easily understandable (n=1088[a])	Female	305 (58.88)	158 (30.50)	33 (6.37)	4 (0.77)	18 (3.47)	.63
		Male	322 (56.49)	176 (30.88)	40 (7.02)	10 (1.75)	22 (3.86)	
		Total	627 (57.63)	334 (30.70)	73 (6.71)	14 (1.29)	40 (3.68)	
	Information clearly laid out (n=1081[a])	Female	241 (47.16)	184 (36.01)	58 (11.35)	12 (2.35)	16 (3.13)	.21
		Male	272 (47.72)	174 (30.53)	84 (14.74)	18 (3.16)	22 (3.86)	
		Total	513 (47.46)	358 (33.12)	142 (13.14)	30 (2.78)	38 (3.52)	
	Texts short and concise (n=1084[a])	Female	124 (24.12)	136 (26.46)	170 (33.07)	59 (11.48)	25 (4.86)	.049
		Male	156 (27.37)	148 (25.96)	158 (27.72)	59 (10.35)	49 (8.60)	
		Total	280 (25.83)	284 (26.20)	328 (30.26)	118 (10.89)	74 (6.83)	
	Visual style/design attractive (n=1076[a])	Female	49 (9.61)	100 (19.61)	192 (37.65)	107 (20.98)	62 (12.16)	<.001
		Male	121 (21.38)	108 (19.08)	164 (28.98)	104 (18.37)	69 (12.19)	
		Total	170 (15.80)	208 (19.33)	356 (33.09)	211 (19.61)	131 (12.17)	
**Website Features**	Possibility to ask questions (n=1079[a])	Female	213 (41.76)	165 (32.35)	83 (16.27)	20 (3.92)	29 (5.69)	.07
		Male	264 (46.40)	142 (24.96)	94 (16.52)	34 (5.98)	35 (6.15)	
		Total	477 (44.21)	307 (28.45)	177 (16.40)	54 (5.00)	64 (5.93)	
	Section with personal experiences (n=1080[a])	Female	176 (34.24)	185 (35.99)	99 (19.26)	32 (6.23)	22 (4.28)	.75
		Male	190 (33.57)	191 (33.75)	113 (19.96)	39 (6.89)	33 (5.83)	
		Total	366 (33.89)	376 (34.81)	212 (19.63)	71 (6.57)	55 (5.09)	
	Advice by other adolescents (n=1072[a])	Female	153 (30.12)	183 (36.02)	112 (22.05)	34 (6.69)	26 (5.12)	.41
		Male	189 (33.51)	184 (32.62)	111 (19.68)	42 (7.45)	38 (6.74)	
		Total	342 (31.90)	367 (34.24)	223 (20.80)	76 (7.09)	64 (5.97)	

[a]Number of participants included in the analysis.
[b]Calculated from chi-square tests.
